# The genetic determinants of renal allograft rejection

**DOI:** 10.1111/ajt.14909

**Published:** 2018-05-28

**Authors:** Maria Hernandez‐Fuentes, Caragh P. Stapleton, Gianpiero L. Cavalleri, Peter Conlon, Michael E. Weale, Graham M. Lord, the United Kingdom and Ireland Renal Transplant Consortium (UKIRTC)

**Affiliations:** ^1^ MRC Centre for Transplantation and NIHR Biomedical Research Centre at Guy's and St Thomas' NHS Foundation Trust and King's College London London UK; ^2^ Department of Molecular and Cellular Therapeutics The Royal College of Surgeons of Ireland and Beaumont Hospital Dublin Ireland

**Keywords:** basic (laboratory) research/science, clinical research/practice, immunogenetics, informatics, kidney (allograft) function/dysfunction, kidney transplantation/nephrology, organ transplantation in general, rejection

*To the Editor:*

We thank Massart et al for their comments^1^ on our recently published large‐scale genome‐wide association study of renal transplant outcomes,^2^ and we welcome the opportunity to examine their findings in more detail.

The 2 recipient genetic loci highlighted in their paper,^3^ rs10765602 (gene annotation *CCDC67*) and rs7976329 (gene annotation *PTPRO*), were well imputed in our study (INFO>0.95) and neither reached genome‐wide significance in our reported analyses.^2^ To provide additional confidence, we have reanalyzed our data following reimputation to the 1000 Genomes phase 3 reference panel via the Sanger Imputation Service (www. imputation.sanger.ac.uk) using Eagle and the Positional Burrows‐Wheeler Transform package.^4^

Table 1 indicates that neither single nucleotide polymorphism (SNP) reaches a nominal level of statistical significance in either donor or recipient genome for our broader definition of acute rejection (any acute rejection event recorded in the first 12 months after transplantation).

**Table 1:** Results from UKIRTC acute rejection GWAS for rs10765602 and rs7976329

Test	rsID	AlleleA	AlleleB	Cases AA	Cases AB	Cases BB	Cases total	ctrls AA	ctrls AB	ctrls BB	ctrls total	MAF cases	MAF controls	*P*	Beta	SE
Recipient (null as missing)	rs10765602	G	T	36	152	209	398	73	319	426	818	0.283	0.284	.702	−0.039	0.102
Recipient (null as missing)	rs7976329	T	C	171	177	50	398	359	359	100	818	0.349	0.342	.636	0.046	0.098
Recipient (null as control)	rs10765602	G	T	36	152	209	398	146	795	941	1881	0.283	0.289	.676	0.039	0.092
Recipient (null as control)	rs7976329	T	C	171	177	50	398	805	839	237	1881	0.349	0.349	.823	0.020	0.087
Donor (null as missing)	rs10765602	G	T	26	160	193	379	49	261	351	661	0.278	0.271	.929	0.010	0.112
Donor (null as missing)	rs7976329	T	C	150	185	44	379	278	300	83	661	0.361	0.352	.361	0.097	0.106
Donor (null as control)	rs10765602	G	T	26	160	193	379	117	623	821	1560	0.278	0.274	.922	−0.010	0.100
Donor (null as control)	rs7976329	T	C	150	185	44	379	656	736	168	1560	0.361	0.343	.323	0.095	0.097

Neither SNP was found to be significantly associated with the acute rejection phenotype in our analysis, in either recipients or donors. The SNPs were imputed to the 1000 Genomes Phase 3 reference panel and analyzed using SNPTEST (frequentist 1, method score). The first 5 principal components, the recruitment site, recipient age, donor age, recipient sex, donor sex, and the total number of HLA mismatches (at A, B, and DR) were included as covariates. Both SNPs, in both recipients and donors, were imputed to a high quality, with an info score and average maximum posterior call of greater than 0.95. Test = recipient/donor to indicate whether recipient or donor genotypes were tested (null as missing /control indicates whether blank records were treated as missing data, or as controls); rsID, SNP identifier; alleleA, noneffect allele (coded as 0); alleleB, effect allele (coded as 1); cases/controls AA, number of case/controls who were homozygous for allele A; cases/controls AB, number of case/controls who heterozygous; cases/controls BB, number of case/controls who were homozygous for allele B; MAF, minor allele frequency (frequency of least common allele in the given dataset); beta, beta coefficient relating to the coded allele (alleleB); SE, standard error of beta coefficient; *P, P‐value*.

The lack of replication signal in our study, despite greater numbers of cases, may be due to a number of factors. We agree with Massart et al that one reason may be the differences in phenotype definition. Our study was primarily designed and powered to detect genetic variation in donor and recipient genomes associated with long‐term graft survival, as this is the key unmet medical need in clinical renal transplantation outcomes, with currently no effective therapeutic options. Our acute rejection phenotype was established from reported national registry‐based outcomes and was not specific to acute T cell–mediated rejection, and thus signal attenuation may be responsible for the difference. However, we note that in our study the recipient minor allele frequency differences between cases and controls are less than 1%, indicating almost complete attenuation. Alternatively, it is possible that the signals found by Ghisdal et al^3^ are false positives. Even genome‐wide significant signals can be false positives, and as the authors used a pooled‐DNA design, and employed a permutation‐based joint test of association and linkage disequilibrium to determine the significance of hits in their discovery phase, it is difficult to determine the combined (discovery + replication) association p‐values for their SNPs. We believe that further data are needed to resolve this issue.

We agree that genetic variation outside the HLA region is an important consideration in seeking to understand the pathogenesis of long‐term graft survival and potentially identifying novel therapeutic targets to reduce cumulative allograft loss over time. We look forward to working with already established international collaborations^5^ to identify these genetic determinants of long‐term graft survival for the benefit of our patients.

## DISCLOSURE

The authors of this manuscript have no conflicts of interest to disclose as described by the *American Journal of Transplantation*.
